# Management of Cardiovascular Disease Patients With Confirmed or Suspected COVID-19 in Limited Resource Settings

**DOI:** 10.5334/gh.823

**Published:** 2020-07-01

**Authors:** Dorairaj Prabhakaran, Pablo Perel, Ambuj Roy, Kavita Singh, Lana Raspail, José Rocha Faria-Neto, Samuel S. Gidding, Dike Ojji, Ferdous Hakim, L. Kristin Newby, Janina Stępińska, Carolyn S.P. Lam, Modou Jobe, Sarah Kraus, Eduardo Chuquiure-Valenzuela, Daniel Piñeiro, Kay-Tee Khaw, Ehete Bahiru, Amitava Banerjee, Jagat Narula, Karen Sliwa

**Affiliations:** 1 Centre for Control of Chronic Conditions, Public Health Foundation India, IN; 2 Department of Non-communicable Disease Epidemiology, London School of Hygiene and Tropical Medicine, GB; 3 All India Institute of Medical Sciences New Delhi, IN; 4 Public Health Foundation of India, IN; 5 World Heart Federation, CH; 6 Pontifical Catholic University of Parana, Curitiba (Brazil), BR; 7 Department of Medicine, Faculty of Clinical Sciences, University of Abuja and University of Abuja Teaching Hospital, NG; 8 Duke Clinical Research Institute, Durham, US; 9 Department of Intensive Cardiac Therapy, National Institute of Cardiology, Warsaw, PL; 10 National Heart Center Singapore and Duke-National University of Singapore, SG; 11 Department of Cardiology, University Medical Center Groningen, University of Groningen, Groningen, NL; 12 Medical Research Council Unit The Gambia, GM; 13 Department of Medicine and the Hatter Institute for Cardiovascular Research in Africa, University of Cape Town, ZA; 14 Radcliffe Department of Medicine, University of Oxford, GB; 15 Instituto Nacional de Cardiología, MX; 16 Universidad de Buenos Aires, AR; 17 Strangeways Research Laboratory, Department of Public Health and Primary Care, Institute of Public Health, University of Cambridge, Worts Causeway, Cambridge, GB; 18 Los Angeles Biomedical Research Institute at Harbor-UCLA Medical Center, US; 19 University College London, GB; 20 Icahn School of Medicine at Mount Sinai|MSSM • Mount Sinai Heart, US; 21 Division of Cardiology, Department of Medicine, Faculty of Health Sciences, University of Cape Town, Groote Schuur Hospital, Cape Town, ZA; 22 Hatter Institute for Cardiovascular Research in Africa, Department of Medicine, Faculty of Health Sciences, Groote Schuur Hospital and University of Cape Town, ZA

**Keywords:** cardiovascular disease, coronavirus, COVID-19, low resource settings, low income countries, middle income countries

## Abstract

In this paper, we provide recommendations on the management of cardiovascular disease (CVD) among patients with confirmed or suspected coronavirus disease (COVID-19) to facilitate the decision making of healthcare professionals in low resource settings. The emergence of novel coronavirus disease, also known as Severe Acute Respiratory Syndrome-Coronavirus-2 (SARS-CoV-2), has presented an unprecedented global challenge for the healthcare community. The ability of SARS-CoV-2 to get transmitted during the asymptomatic phase and its high infectivity have led to the rapid transmission of COVID-19 beyond geographic regions, leading to a pandemic. There is concern that COVID-19 is cardiotropic, and it interacts with the cardiovascular system on multiple levels. Individuals with established CVD are more susceptible to severe COVID-19. Through a consensus approach involving an international group this WHF statement summarizes the links between cardiovascular disease and COVID-19 and present some practical recommendations for the management of hypertension and diabetes, acute coronary syndrome, heart failure, rheumatic heart disease, Chagas disease, and myocardial injury for patients with COVID-19 in low-resource settings. This document is not a clinical guideline and it is not intended to replace national clinical guidelines or recommendations. Given the rapidly growing burden posed by COVID-19 illness and the associated severe prognostic implication of CVD involvement, further research is required to understand the potential mechanisms linking COVID-19 and CVD, clinical presentation, and outcomes of various cardiovascular manifestations in COVID-19 patients.

## Introduction

In late 2019, an outbreak of viral pneumonia was reported in Wuhan, Hubei province, China. Caused by a new coronavirus called SARS-COV-2 (initially 2019-nCOV), the infection spread from a local seafood market where wild animals, such as bats –the possible reservoir of the virus, were sold [1]. Although initial cases may have been infected through zoonotic exposure, infection spread through human-to-human transmission, mainly via respiratory droplets and close interpersonal contact with those infected, including asymptomatic carriers [2,3]. On average, each carrier transmits the disease to 2-3 other people [4,5,6]. The incubation period is four to five days but may reach 14 days [6]. Affected patients have fever, dry cough, dyspnea, headache and pneumonia [7], in a similar clinical picture of those affected by the SARS epidemic in 2003 [8]. Atypical features include a low prevalence of sneezing and gastrointestinal symptoms including nausea in about a third of patients.

**Figure 1:**
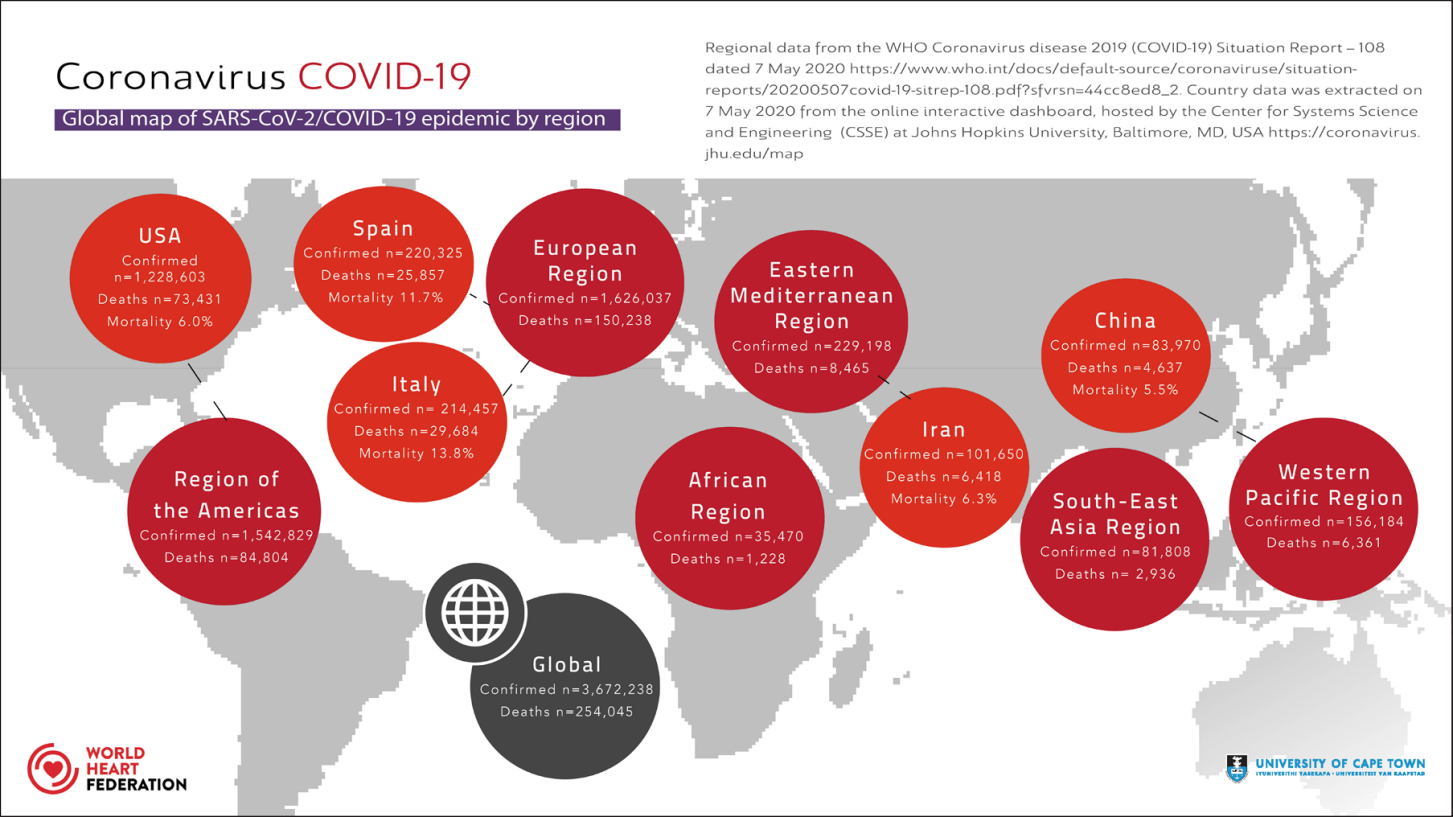
Global Map of COVID-19 pandemic by region.

This new disease, now called COVID-19 [9], was declared a pandemic by the World Health Organization (WHO) on March 11^th^. To date, COVID-19 has spread to 212 countries/ areas/ or territories, affected 3, 672, 238 persons and resulted in the death of 254, 045 patients (Figure 1) [10], with substantially growing numbers of infections and deaths as the pandemic progresses. Although 80% of all patients with confirmed COVID-19 may have only mild or moderate symptoms, the case-fatality ratio is highly variable depending on how the cases are defined, the testing practices, the health system access and other unknown factors. Reports provide a range between 2-7% overall and may be as high as 20% in the elderly [11].

The interaction between COVID-19 and the cardiovascular system has been a subject of special attention.

Patients with pre-existing cardiovascular disease (CVD), hypertension, or diabetes mellitus appear to be at increased risk of having severe form of the disease. The involvement of the cardiovascular system had already been described in other epidemics caused by corona viruses [12]. In COVID-19, patients with or at risk for CVD seem to be at a higher risk of more severe disease, the need of intensive care unit (ICU) [13], and mortality [14,15].

There are different potential mechanisms suggested for the link between COVID-19 and CVD. The potential pathophysiological links between COVID-19 and CVD include virus-induced inflammation, increase risk of thrombosis, ischemia due to increased oxygen demand, microvascular ischemic injury and the accelerated immunologic response (cytological storm) mediated injury [16].

Also, there have been reports of cardiac complications in COVID-19 patients including myocardial injury, acute coronary events and heart failure. It has been reported that patients with these complications (including troponin elevation) are at a higher risk of mortality [17].

Different organizations have issued recommendations for the management of patients with cardiovascular disease at the time of COVID-19 pandemic [18,19,20].

The management of more seriously ill patients with COVID-19 requires resources, like medical supplies, ventilators, personal protective equipment (PPE), that could become limited, even in high-income countries. Dealing with this situation in countries where resources are scarce is even more challenging. In the context of COVID-19, low resource setting refers to a setting with limited capacity to diagnose, quarantine, isolate, and treat COVID-19 patients and the cardiovascular complications or their illness, as well as acute coronary syndromes, including the use of invasive diagnostic or treatment support for acute coronary syndromes in both COVID-19 positive and negative patients.

The unprecedented burden of COVID-19 on health systems threatens to exceed hospital capacity, making it challenging to care for other emergencies requiring hospitalization, such as acute coronary syndromes (ACS).

In order to support WHF members, particularly those working in low-resource settings, a writing group including members of the WHF Science Committee and WHF Emerging Leaders under the leadership of the Chair of the Science Committee and WHF President developed a statement with recommendations to manage patients with CVD and COVID19. It is important to emphasize that this document is not a clinical guideline and should not replace established national clinical guidelines and recommendations nor should override the individual responsibility of health care professionals when managing patients with these conditions.

To develop this document, we looked at relevant guidelines, reports and recommendations and through a consensus process we agreed on key recommendations on the following topics: general recommendations, hypertension and diabetes, acute coronary syndrome, heart failure (including Rheumatic Heart Disease and Chagas Disease), and myocarditis.

## General recommendations

COVID-19 patients need to be triaged based on disease severity with patients with moderate and severe disease admitted in segregated ward/hospital depending on the available infrastructure. These patients also need to be triaged based on co-morbidities like hypertension, diabetes, prior cardiovascular or respiratory disease, renal failure and cancer to identify patients with a higher likelihood to have severe disease. Below are some broad guidelines with particular reference to low resource settings.

## 1. Hypertension and diabetes management in COVID-19 positive patients

The cardiovascular manifestations of severe acute respiratory syndrome corona virus -2 (SARS-CoV 2) infection remain under investigation, however, early evidence from several epidemiological studies suggests that patients with CVD or risk factors including hypertension and diabetes have up to a 3-fold increased risk for severe morbidity and mortality [18,19,20]. Early genomic studies into the pathogenesis of cardiovascular involvement in COVID 19 infection have indicated a possible mechanism involving the angiotensin-converting enzyme 2 (ACE-2) receptor which is expressed in epithelial cells in the lungs, heart, intestines, and kidneys [21,22,23,24,25,26].

The SARS CoV-2 binds ACE-2 receptors expressed on epithelial tissues and uses that as its main functional component to gain entry to lungs and myocardium – a hypothesis largely formulated based on animal models [21,22,23,24,25,26]. ACE-2 is an important component of the renin-angiotensin-aldosterone system (RAAS) which cleaves angiotensin-II – a vasoconstrictor into angiotensin – a vasodilator. It serves to counterbalance the effects of angiotensin converting enzyme (ACE) which converts angiotensin I hormone into angiotensin II. Several prior studies have shown that angiotensin converting enzyme inhibitors/angiotensin II receptor blockers (ACE-i/ARB) lead to upregulation of ACE-2 enzyme therefore enhancing vasodilatory effects of renin and angiotensin blockade treatments [21,22,23,24,25,26]. Therefore, theoretically the upregulation of ACE2 activity in COVID-19 patients on RAAS treatment may create a higher functional substrate for the virus to bind to and cause severe organ damage. However, this is not borne out in observational studies published recently [27].

In a recent analysis from Lombardy region of Italy that compared 6,272 people with confirmed SARS-CoV-2 infection with 30,759 controls matched by age, sex, and municipality of residence [28,29]. It was shown neither ACE inhibitors nor ARBs were associated with the probability of SARS-CoV-2 infection [28].

A review based on a database analysis of an international registry from 169 hospitals located in 11 countries in Asia, Europe, and North America studied the effects of treatment on outcomes of these patients who had either died in the hospital or survived to hospital discharge among 8,910 patients of COVID-19 [30]. It was revealed in this analysis that age greater than 65 years, coronary artery disease, congestive heart failure, history of cardiac arrhythmia, chronic obstructive pulmonary disease, and current smoking were associated with an increased risk of in-hospital death. Female gender was associated with better outcomes. Furthermore, neither ACE inhibitors nor ARBs were associated with an increased risk of in-hospital death. A secondary analysis that was restricted to patients with hypertension (those for whom an ACE inhibitor or ARB would be indicated) also did not show harm.

Based on available evidence we recommend that treatment with ACE inhibitors and ARBs should be continued in patients receiving it for hypertension management. This is in line with a similar recommendation by other cardiac societies like ACC and ESC.

Diabetes has also been identified as a significant comorbid condition associated with worse outcomes in COVID-19 infected patients [19,20,31]. In general, there is a consensus that good glycemic control both in hospitalized and non-hospitalized COVID-19 infected patients is key to reduce severe morbidity and mortality from the infection like studies from other systemic infections including influenza [19,31]. Thus, guidelines for diabetes management and glycemic control measures as before should be followed.

## 2. Acute Coronary Syndrome

An ACS diagnosis should be based on history, the electrocardiogram (ECG), and troponin levels. However, classic symptoms and presentation of ACS may be overshadowed in the context of COVID-19, potentially leading to under-diagnosis. Because troponin elevation may be present at presentation in 7%–28% [17,20,32,33,34] of patients with COVID-19, even in the absence of an ischemic etiology, the clinical history and ECG must be considered in the interpretation of troponin results. It is also essential to have serial troponin measurements to determine whether the myocardial injury reflected by the elevated troponin value is acute or chronic.

The rule should be ACS treatment according to the guidelines. Even rapid COVID-19 tests cannot delay ST-segment elevation myocardial infarction (STEMI) treatment. Each patient must be treated as potentially infected. Special precautions should apply to patients with high fever and contact with infected COVID-19. It needs to be emphasised that facilities must have separate facilities in place for dealing with COVID-19 cardiac patients and non COVID-19 cardiac patients including catheterization laboratories.

**Overarching issues**

- A dedicated infrastructure and process are needed for management of ACS in patients with COVID-19. Invasive management should be reserved for critical patients provided that the facility has systems in place for non-transmission of coronavirus during transport and treatment.
- If existing hospitals have cardiac units with more than one catheterization laboratory, one lab could be designated for management of COVID-19 positive or suspected COVID-19 patients. The catheterization lab needs proper terminal cleaning after managing a COVID-19 patient.
- Information on where management is available for suspected or diagnosed COVID-19 patients with ACS must be clearly circulated. Testing for diagnosis of COVID-19 must be available at the facilities managing the ACS patients.

Invasive management; any type of catherization procedure – diagnostic or intervention – if the patient is hemodynamically stable is deferred.

Reperfusion therapy in ACS (e.g. STEMI) should consider the clinical presentation, risk to treating medical personnel, staff availability and the availability of high dependency beds in a hospital. Patients with coronary artery disease may be at increased risk because of coronary plaque rupture secondary to virally induced systemic inflammation. Use of plaque stabilizing agents (aspirin, statins, beta-blockers, and angiotensin-converting enzyme inhibitors) has been suggested as a possible therapeutic strategy. Anti-platelet therapy may be advised to treat the pro-coagulant effects of systemic inflammation that may increase the likelihood of stent thrombosis.

### Current recommendations for ACS management

Confirmed COVID-19 infections:

- STEMI: Low risk STEMI patients, consider thrombolysis as the treatment of choice. Cardiac catheterization should be considered only for rescue PCI.
- STEMI: High risk STEMI patients. The risks to the treating personnel should be considered before deciding on primary PCI. If PPE is available and the hospital cath lab personnel are well versed in its use, then consider primary PCI. In all other situations, thrombolysis should be the treatment of choice.
- Low risk NSTEMI: Conservative management.

Suspected COVID-19 infection presenting with ACS:

- STEMI: Thrombolysis should be the reperfusion strategy of choice, like in patients with confirmed COVID-19 cases.
- NSTEMI: Conservative management until the confirmatory test results are available. Timing should allow for diagnostic testing for COVID-19 before cardiac catheterization and allow for a more informed decision regarding infection control.
  - Patients with COVID-19 can have significant thrombocytopenia. This should be considered when deciding the revascularisation strategy [32].

**Figure 2:**
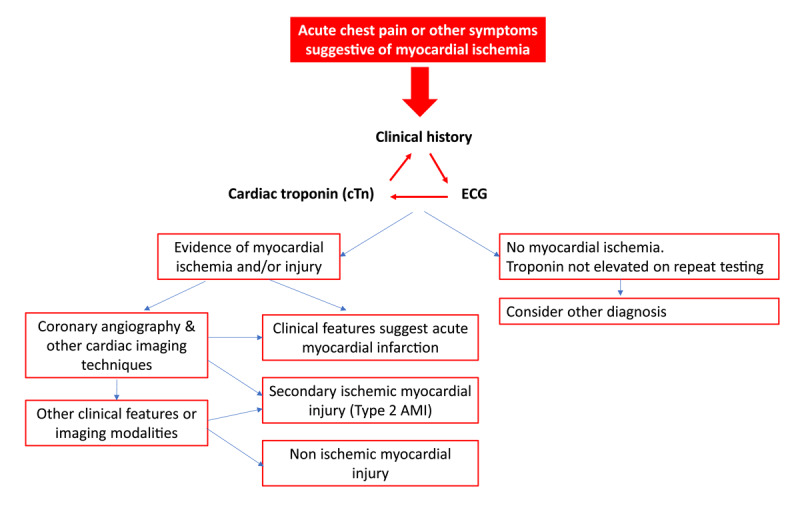
Outcomes of acute chest pain [35]. Figure 2 adapted from ACC guideline Diagnosing Type 2 Myocardial Infarction [35].

Figure 2 summarizes the overall management of acute chest pain.

## 3. Myocardial injury

**Figure 3:**
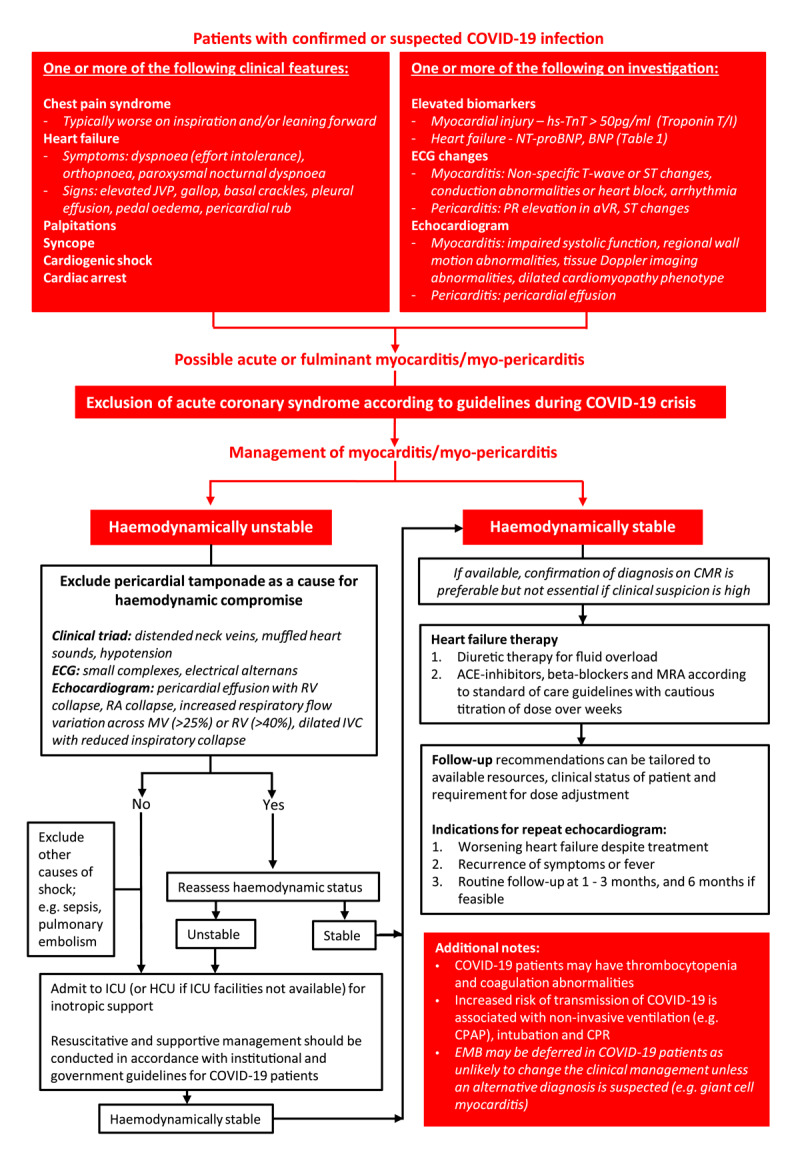
Diagnosis and management of myocardial injury in COVID-19 patients. **Legend:** ACE, angiotensin converting enzyme; BNP, brain natriuretic peptide; CPAP, continuous positive airway pressure; ECG, electrocardiogram; EMB; endomyocardial biopsy; HCU, high care unit; hs-TnT, high sensitivity troponin T; ICU, intensive care unit; JVP, jugular venous pressure; MRA, mineralocorticoid receptor antagonists; MV, mitral valve; RA, right atrium; RV, right ventricle; TV, tricuspid valve.

Myocardial injury, diagnosed by raised troponin is common in patients with moderate to severe COVID-19 and is associate with poor prognosis. Although, myocarditis was initially proposed as a mechanism, it is increasingly clear that there is minimal inflammation on biopsy and autopsy studies. There are no viral particles in myocardium even though there is abundance of ACE2 receptors on myocardium and coronary vascular endothelium. The viral particles demonstrated in myocardium are contained within macrophages which have arrived from lungs. The overwhelming cause of injury in absence of CAD is proposed to be systemic cytokine response, micro vascular coagulation and demand ischemia. Cytokine storms lead to increased vascular permeability and myocardial oedema with transient damage to the myocardium [36,37,38]. Chinese expert consensus statements report that immunological damage is the predominant mechanism observed in adult patients and is associated with a more rapid recovery of cardiac function [39]. Figure 3 outlines approach to a patient with myocardial injury.

**ECG in Patients with COVID-19:** Sinus tachycardia is a common but non-specific finding in COVID-19 patients and may be present in the absence of myocardial injury. Non-localizing ST changes (typically concave in shape) and/or non-specific T-wave changes are suggestive of underlying myocardial involvement. New conduction abnormalities, such as atrioventricular block, sinus arrest and QRS prolongation, and arrhythmias (atrial or ventricular) are suggestive of underlying cardiac involvement [40].

**Chest Radiograph:** Radiographically, it may be difficult to distinguish pulmonary oedema from viral pneumonia, however, some distinguishing features may be useful (Table 1). Radiographic findings should always be interpreted in conjunction with other clinical findings. Clinical or radiological improvement after administration of diuretics may support the presence of pulmonary oedema.

**Cardiac structural and functional abnormalities:** Ventricular dysfunction (systolic or diastolic) with or without dilatation of the ventricles indicates a significant myocardial insult. Regional wall motion abnormalities may be present and are supportive of myocarditis particularly when observed at rest and seen in combination with non-localizing ST changes on ECG and a positive troponin. Viral myocarditis frequently affects the basal infero-lateral wall. Additional features that may be present include, increased ventricular wall thickness, pericardial effusion and intracardiac thrombus formation [40].

**Table 1:** Chest radiographic findings in COVID-19 pneumonia and cardiogenic pulmonary oedema.

COVID-19 Pneumonia	Cardiogenic Pulmonary Oedema

**Typical:** – Peripheral bilateral ground glass opacities (GGO) **with or without** consolidation – Multifocal GGO with rounded morphology	**Typical (acute heart failure):** – Central, peri-hilar bilateral GGO with peripheral sparing (‘batwing distribution’) – Interlobular septal thickening (Kerley B lines) – Pleural effusions – Peri-bronchial cuffing **Additional features seen in chronic heart failure:** – Upper lobe blood diversion – Azygos distension – Cardiomegaly
**Intermediate:** Absence of typical features AND the presence of: – Multifocal, diffuse, perihilar or unilateral GGO with or without consolidation	
**Atypical:** – Isolated lobar or segmental consolidation without GGO – Smooth interlobular septal thickening with pleural effusions	**Atypical:** – Unilateral GGO, with or without pleural effusions – Pulmonary pseudotumour (fluid within the interlobar fissure)

Courtesy of Vishesh Sood, Department of Radiology, Groote Schuur Hospital, South Africa. Adapted from Simpson et al. Radiology: Cardiothoracic Imaging. 2020; 2(2): e200152 [41] and Gluecker et al. Radiographics. 1999; 19(6):1507–1531 [42].

### Management of myocardial injury in COVID-19 patients

Management of COVID-19 related myocardial injury is outlined in Figure 3. Current guidelines for the treatment of viral myocarditis should be applied, including the use of standard heart failure therapies and supportive measures [40,43]. Prednisolone has shown some benefit in a few isolated case reports, however, there is insufficient evidence to support the routine use of steroids in these patients and may cause harm [44].

### Thrombosis and anticoagulation

COVID-19 infection has been associated with pro-thrombotic abnormalities [45]. Studies have shown that patients with COVID-19 might present with increased D-dimer levels, fibrin degradation products, and prothrombin time (PT) prolongation; all changes that are associated with a higher risk of poor outcome [46]. These hemostatic changes indicate some forms of coagulopathy that may predispose to thrombotic events, but whether these changes are a specific consequence of SARS-CoV-2 or a result of systemic inflammatory response syndrome (SIRS) is still uncertain [47].

As any hospitalized patients with an acute medical illness, patients with COVID-19 are at increased risk of venous thrombotic events and prophylactic anticoagulation is indicated to reduces their risk as indicated by clinical guidelines [48].

Based on the hemostatic changes observed in COVID-19, it has been hypothesized that anticoagulation (rather than prophylaxis) could be beneficial for patients with COVID-19. The evidence is only emerging, and there is some data showing that anticoagulation could indeed be beneficial [49,50]. However, the evidence so far is based on sub-group analyses of observational studies and further randomised controlled studies are needed to confirm or refute this hypothesis.

### Follow-up

There is currently no data available on the long-term sequelae of COVID-19 myocarditis. Current recommendations for the management of other forms of viral myocarditis should be followed in the interim [40]. It is recommended that patients with structural and functional abnormalities have a repeat echocardiogram in 1-3 months after discharge and are followed up for a minimum of six months. Where left ventricular (LV) recovery is delayed beyond three months, heart failure therapy should be continued for a minimum of six months to a year. Long-term therapy heart failure therapy is advised in patients with persistent LV dysfunction, conduction abnormalities, or those with evidence of myocardial scar.

## 4. Heart failure and COVID-19

Case fatality rate in patients with CVD including heart failure has been reported to be as high as 10.5% compared to fatality rate of 2.3% in the general population [51].

The management of heart failure (HF) patients with suspected or confirmed COVID-19 begins with recognizing that respiratory infection is a common trigger of HF decompensation. Patients with chronic cardiac conditions (including HF) are predisposed to respiratory infections and their complications with some symptoms and signs of both cardiac and respiratory conditions overlapping. An overarching consideration in the management of such patients is the very high virulence and transmissibility of COVID-19, necessitating extraordinary efforts to minimize exposure for both patients and medical staff.

### Guidance during clinical evaluation and management

Stable patients with chronic heart failure (HF) may be followed up via telemedicine as far as possible, with elective procedures deferred, thus minimising their exposure to infection. Routine flu vaccination should be considered for these patients with chronic HF.

In unstable patients, presenting to the healthcare facility, protection to healthcare staff and other patients should be ensured during clinical evaluation. This includes adequate physical distancing, proper application of PPE for both staff and patients, segregation of staff, wards and equipment as far as possible. For example, patient front-facing versus non-patient-facing teams, COVID-19 positive versus negative patients wards to minimize cross-contamination, thorough disinfection of equipment used for patient evaluation, restricting visitors and using virtual means to communicate with patients’ relatives as much as possible [52,53].

Clinical assessment should focus on history and physical examination, isolating patients until the diagnosis of COVID-19 is established or excluded and this may require repeated swab testing in cases of high pretest probability. Standard investigations in the evaluation of HF patients [54,55] should be considered with the following caveats:

1. Request only tests that are expected to change management or are potentially lifesaving.
2. Conduct assessment at the patient’s bedside as much as possible.
3. Circulating biomarkers, for example, natriuretic peptides and cardiac troponins that may facilitate differential diagnoses or risk stratification for further investigations should be prioritised.
4. Echocardiography where indicated should be conducted using a focussed protocol [52] and by point-of-care ultrasonography where available.*
5. In hemodynamically and electrically stable patients, routine angiography or advanced cardiac imaging may not be necessary or can be delayed.
6. Routine endomyocardial biopsy in patients with active COVID-19 is discouraged.

Echocardiography should be used with adequate precautions for person and equipment to maintain barrier based on resources available. Considerations should focus on ‘Whom to Image’, ‘Where to Image’ and ‘How to Image’ with the aim to restrict transmissions [56].

Treatment of patients with heart failure and confirmed COVID-19 should include the following considerations:

1. Establish the patient goals of care via effective doctor-patient communication, for example e.g. preference for cardiopulmonary resuscitation and ventilation.
2. Continuation of HF guideline-directed medical therapies is generally warranted despite controversies regarding the effect of angiotensin converting enzyme inhibitors and angiotensin receptor blockers on COVID-19 (coronaviruses gain entrance to cells via the ACE2 receptor, which might be upregulated by use of these therapies). Despite this, all major cardiology societies have recommended against the withdrawal of renin-angiotensin system inhibitors based on available data unless otherwise clinically indicated.
3. A multi-disciplinary team approach is encouraged, including an infectious disease consultant and intensive care specialist and pulmonologist.
4. Given limited high-quality evidence and lack of universally accepted guidelines specific to the management of COVID-19-related cardiac disease, enrolment in ongoing clinical trials is encouraged.
5. Among experimental approaches.
  - Hydroxychloroquine has been proposed to treat COVID-19 based on in-vitro data and a small open-label study with significant methodological limitations, however further studies are warranted. As mentioned above, it should be noted that hydroxychloroquine could prolong QT interval and cause arrhythmias.
  - Antiviral therapies especially hold promise but need further study.
  - Immunosuppression has been used as salvage therapy in selected cases, however given concerns that steroids may prolong viral persistence, steroid treatment is not routinely recommended in all cases.
  - Anti-inflammatory agents e.g. interleukin-6 inhibitors are under investigation in patients with severe COVID-19.
  - Convalescent plasma from recovered COVID-19 patients has been approved by the FDA and warrants further study.
6. Mechanical circulatory support may be indicated in selected patients with refractory shock.
7. Patients who recover from acute episodes should be closely followed up for any longer-term sequelae.

## Other heart failure and COVID-19 considerations relevant in low resource settings

### Rheumatic Heart disease

At this current stage there are no data on the vulnerability of patients with rheumatic heart disease (RHD) and the impact of COVID-19 infection. Patients with RHD are usually between ages 20s and 30s and may have left ventricular dysfunction or pulmonary hypertension making them more vulnerable to the complicated COVID disease. Patients should be made aware of the consequences of infection and advised to observe social distancing. They should continue their usual medication even where they experience suspicious symptoms for COVID-19 while awaiting test results. The World Heart Federation COVID cardiovascular disease survey (WHF-COVID) is planned to commence May 2020 and will hopefully provide much needed data.

### Chagas disease

There is limited information available about the risk of stable asymptomatic patients, therefore people with chagas disease should follow the same recommendations as general population. However, those with complicated Chagas disease do have a higher risk because of the high prevalance of heart failure, as previously mentioned linked to higher risk of complications in patients with COVID-19. A decision should be made as to whether to suspend antiparasitic treatment and recommence once recovered from COVID-19.

Summary of Chagas disease severity and treatment recommendation:

1. Indeterminate form or without apparent cardiopathy.
  - Risk comparable to the general population;
  - Suspension or delay of antiparasitic drugs if symptomatic COVID-19.
2. Chagas cardiopathy without heart failure.
  - Higher arrhythmic risk (even if asymptomatic);
  - Cautious use of medications that produce arrhythmias;
  - Suspension or delay of antiparasitic drugs if symptomatic COVID-19.
3. Chagas cardiopathy with heart failure.
  - Higher risk of death or complications related to the COVID-19;
  - Higher arrhythmic risk;
  - No indication for antiparasitic drugs use.

### Final comments

The WHF Science Committee will continue to closely monitor the evolving nature of the data on COVID-19 and its potential link with cardiovascular diseases. We strive to keep updating the latest information on the clinical implications of COVID-19 outbreak on cardiovascular diseases and organize webinars involving expert panelists to share experience of the front-line health workers managing COVID-19 and cardiovascular diseases. Furthermore, WHF COVID-19 CVD global survey will provide key insights to inform clinical and policy practices, for a better understanding of the cardiovascular conditions that increase the risk of developing severe COVID-19, and a better characterization of cardiovascular complications in hospitalized patients with COVID-19.
