# Correction: Management of Cardiovascular Disease Patients With Confirmed or Suspected COVID-19 in Limited Resource Settings

**DOI:** 10.5334/gh.885

**Published:** 2020-08-07

**Authors:** Dorairaj Prabhakaran, Pablo Perel, Ambuj Roy, Kavita Singh, Lana Raspail, José Rocha Faria-Neto, Samuel S. Gidding, Dike Ojji, Ferdous Hakim, L. Kristin Newby, Janina Stępińska, Carolyn S. P. Lam, Modou Jobe, Sarah Kraus, Eduardo Chuquiure-Valenzuela, Daniel Piñeiro, Kay-Tee Khaw, Ehete Bahiru, Amitava Banerjee, Jagat Narula, Fausto J. Pinto, David A. Wood, Karen Sliwa

**Affiliations:** 1 Centre for Control of Chronic Conditions, Public Health Foundation India, IN; 2 Department of Non-communicable Disease Epidemiology, London School of Hygiene and Tropical Medicine, GB; 3 All India Institute of Medical Sciences New Delhi, IN; 4 Public Health Foundation of India, IN; 5 World Heart Federation, CH; 6 Pontifical Catholic University of Parana, Curitiba (Brazil), BR; 7 Department of Medicine, Faculty of Clinical Sciences, University of Abuja and University of Abuja Teaching Hospital, NG; 8 n/a; 9 Duke Clinical Research Institute, Durham, US; 10 Department of Intensive Cardiac Therapy, National Institute of Cardiology, Warsaw, PL; 11 National Heart Center Singapore and Duke-National University of Singapore, SG; 12 Department of Cardiology, University Medical Center Groningen, University of Groningen, Groningen, NL; 13 Medical Research Council Unit, GM; 14 Department of Medicine and the Hatter Institute for Cardiovascular Research in Africa, University of Cape Town, ZA; 15 Radcliffe Department of Medicine, University of Oxford, GB; 16 Instituto Nacional de Cardiología, MX; 17 Universidad de Buenos Aires, AR; 18 Strangeways Research Laboratory, Department of Public Health and Primary Care, Institute of Public Health, University of Cambridge, Worts Causeway, Cambridge, GB; 19 Los Angeles Biomedical Research Institute at Harbor-UCLA Medical Center, US; 20 University College London, GB; 21 Icahn School of Medicine at Mount Sinai|MSSM • Mount Sinai Heart, US; 22 Faculty of Medicine at the University of Lisbon, PT; 23 Imperial College London, GB; 24 Division of Cardiology, Department of Medicine, Faculty of Health Sciences, University of Cape Town, Groote Schuur Hospital, Cape Town, ZA; 25 Hatter Institute for Cardiovascular Research in Africa, Department of Medicine, Faculty of Health Sciences, Groote Schuur Hospital and University of Cape Town, ZA

## Abstract

This article details a correction to the article: Prabhakaran D, Perel P, Roy A, Singh K, Raspail L, Faria-Neto JR, et al. Management of Cardiovascular Disease Patients With Confirmed or Suspected COVID-19 in Limited Resource Settings. *Global Heart*. 2020; 15(1): 44. DOI: http://doi.org/10.5334/gh.823

## Correction

After the publication of ‘Management of Cardiovascular Disease Patients With Confirmed or Suspected COVID-19 in Limited Resource Settings.’ [1], the editors informed the publisher that two of the co-authors, Fausto J. Pinto and David A. Wood, had been accidentally left off the author list. The author list has been corrected here to show the full list of authors.
